# Estimating the population impact of hypothetical breastfeeding interventions in a low-income population in Los Angeles County: An agent-based model

**DOI:** 10.1371/journal.pone.0231134

**Published:** 2020-04-09

**Authors:** Linghui Jiang, Xiaoyan Li, May C. Wang, Nathaniel Osgood, Shannon E. Whaley, Catherine M. Crespi

**Affiliations:** 1 Department of Community Health Sciences, Fielding School of Public Health, University of California, Los Angeles, California, United States of America; 2 Department of Computer Science, University of Saskatchewan, Saskatoon, Canada; 3 Public Health Foundation Enterprises, Special Supplemental Nutrition Program for Women, Infants, and Children (PHFE-WIC) Program, Los Angeles, California, United States of America; 4 Department of Biostatistics, Fielding School of Public Health, University of California, Los Angeles, California, United States of America; Monash University, AUSTRALIA

## Abstract

**Background:**

Breastfeeding has clear benefits. Yet, breastfeeding practices fall short of recommendations in low-income populations including participants of the Special Supplemental Nutrition Program for Women, Infants, and Children (WIC). To promote breastfeeding, it is important to understand breastfeeding-related behaviors such as initiation and maintenance within the context of a complex societal system. For individual women, making choices about infant feeding (whether to breastfeed or formula-feed a newborn, or when to stop breastfeeding) is a dynamic process involving interactions with health professionals, family, peers and workplaces. Integrating behavioral change theories with systems science tools such as agent-based modeling can help illuminate patterns of breastfeeding behaviors, identify key factors affecting breastfeeding behaviors within this complex dynamic system, and estimate the population impact of hypothetical interventions.

**Methods:**

An agent-based model (ABM) was developed to investigate the influences of multiple levels of factors affecting breastfeeding behaviors among WIC participants. Health behavioral change theories were applied and stakeholder input obtained to improve the model, particularly during the conceptual design and model specification steps. The model was then used to identify critical points for intervention and assess the effects of five common interventions (improving knowledge through education, implementing Baby-Friendly Hospital Initiative practices, providing postpartum breastfeeding counselling, strengthening partner support, and fostering supportive workplace environments.)

**Results:**

The ABM developed in this study produced outcomes (i.e., breastfeeding rates) that were concordant with empirical data. Increasing the coverage of the five selected interventions produced various levels of improvement in breastfeeding practices in the target population. Specifically, improving breastfeeding knowledge had a positive impact on women’s *intent to* breastfeed, while increasing the availability of the Baby-Friendly Hospital Initiative improved breastfeeding *initiation* rates. However, neither of these two interventions showed a significant impact on breastfeeding *maintenance*, which was supported by postpartum breastfeeding counseling, partner support and a supportive workplace environment. These three intervention strategies each improved breastfeeding rates at 6 months from 55.6% to 57.1%, 59.5% and 59.3%, respectively. Increasing the coverage of multiple interventions simultaneously had a synergistic effect on breastfeeding *maintenance* with their effects being greater than the cumulative effects of increasing the coverage of these interventions individually.

**Conclusion:**

The ABM we developed was helpful for understanding the dynamic process of decision-making regarding infant feeding modalities in a low-income population, and for evaluating the aggregated population-level impact of breastfeeding promotion interventions.

## Introduction

Breastfeeding has many health and other benefits for both mother and baby and is recommended as the optimal feeding practice for infants, world-wide [1–3]. In the United States, low-income women are less likely to breastfeed and meet the recommended breastfeeding duration [4]. For example, women from households with income less than 100% federal poverty level have breastfeeding rates that are 24% lower at 6 months and 26% lower at 12 months than the national average [4]. Considerable efforts have been made by the Special Supplemental Nutrition Program for Women, Infants, and Children (WIC), a major federal nutrition assistance program for low-income families, to promote breastfeeding. While a few studies have reported on evaluations of breastfeeding promotion programs in the WIC population [5–8], more translational research is needed to determine which intervention strategies are most effective.

The socioecological framework [9] can be applied to help us understand how individual, interpersonal, and societal/structural level factors interact to influence breastfeeding behaviors, namely, a mother’s decision to initiate, maintain or stop breastfeeding. These factors include knowledge and education at the individual level, family and peer support at the interpersonal level, and social norms and workplace policies at the societal/structural level [10]. Importantly, these factors are not independent of each other–a mother’s breastfeeding behavior reflects a dynamic process featuring learning and adaptation through interactions with others and the environment; at the same time, her behavior may also influence others and the environment.

The fact that a mother’s breastfeeding practices are embedded in such a complex system presents considerable challenges for predicting the potential effects of certain interventions (e.g., educational and workplace support programs) and selecting the most effective intervention strategies. Traditional analytic methods that assume independence (of measurements/data points) and static effects may not capture the dynamic interactions among the various factors. Practical and ethical constraints also render the application of experiments to evaluate the impact of ‘real world’ interventions almost impossible.

Over the past decade, the National Institutes of Health have encouraged the application of systems science methods such as agent-based modeling in public health research to advance our understanding of causality regarding health conditions and facilitate breakthroughs to improve population health [11]. Agent-based modeling is “a computational method that enables a researcher to create, analyze, and experiment with models composed of agents that interact within an environment” [12]. In an agent-based model, individual entities (agents) and their interactions with each other and with their environment are directly represented. Compared to traditional variable-based statistical equations, agent-based modeling methods hold several advantages [12–14]. First, they allow researchers to model heterogeneous individuals (agents) and the dynamic interactions among agents in a complex system (say, a breastfeeding mother and her interactions with family, healthcare, and co-workers). Second, they allow the agents in the model to ‘adapt and learn’ as they do in reality. Such models, therefore, are able to capture history-dependent behaviors. For example, breastfeeding later is not possible if one has stopped earlier, or never initiated in the first place. Third, behavioral theories about process can be relatively easily represented in the model through explicit decision rules for individual actions. Fourth, agent-based models represent multiple levels of analysis in a natural way that allows for the investigation of the aggregated effects of interventions at the population level that result from individual decision-making and practices. Fifth, agent-based models allow us to set up and run experiments using various input values (parameters) in order to study the possible outcomes of hypothetical interventions and answer many “what if …” policy questions. Lastly, the visual presentation of results produced by an agent-based model serves as an effective communication tool for disseminating research findings and influencing policy decisions.

Agent-based modeling methods have long been used in other disciplines and are increasingly used in public health research [12, 14, 15]. Some recent investigations applying this analytical tool in the field of public health suggest that it holds promise for investigating causal mechanisms of health problems and evaluating the effects of policy and program interventions [16–19]. However, these models are mostly built on single decision rules or focus on a single intervention. Agent-based models that examine complex behaviors by incorporating multiple influencing factors from various levels have not been fully explored.

The objective of this study is to develop an ABM for investigating the multiple factors that influence breastfeeding practices, and to use the model to evaluate the impact of a set of hypothetical interventions in the WIC population in Los Angeles County. The specific aims are to: (1) build an agent-based simulation model which incorporates behavioral theories and includes individual and environmental factors that may influence breastfeeding behaviors in a low-income population; and (2) estimate the population impact of a set of hypothetical interventions on breastfeeding to inform selection of effective intervention strategies for promoting breastfeeding in a low-income population.

## Methods

This study developed an ABM using the simulation software AnyLogic (version 8.3.2). The ABM represents infant feeding decisions and practices of a cohort of low-income women during the first 6 months postpartum. This study focuses on modeling the breastfeeding practices of primiparous women. Primiparous women have significantly different breastfeeding experiences than multiparous women; and the breastfeeding experience of the first child is closely associated with breastfeeding practices for subsequent births [20, 21]. The ABM is used to identify critical points for intervention and to assess the effects on breastfeeding of several common interventions (improving knowledge, implementing Baby-Friendly Hospital Initiative practices, providing postpartum breastfeeding counselling, strengthening partner support, and fostering a supportive workplace environment) at the population level. The following section provides details about each step of the model building and testing process. A supplement provides additional detailed information.

### Model scope and conceptual design

**Fig 1:**
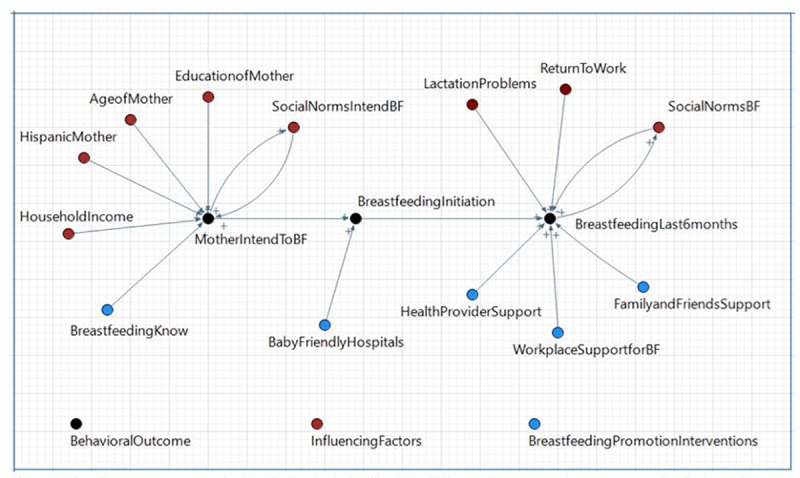
Conceptual framework for the breastfeeding agent-based model.

We simulated a cohort of primiparous women with different socio-demographic characteristics and modeled their breastfeeding experience during the first 6 months postpartum using the conceptual framework shown in Fig 1. The development of this framework was informed by a literature review of key health behavioral change theories that apply to breastfeeding and consultation with content experts, including a professional lactation expert, a pediatrician, and a nutritionist.

The conceptual framework, in the form of a causal loop diagram (Fig 1), captures three key stages of the breastfeeding behavioral process–intent to breastfeed (during pregnancy), initiation of breastfeeding (following childbirth), and maintenance of breastfeeding through 6 months (postpartum)–and key factors influencing breastfeeding practices at each stage, including breastfeeding promotion interventions. During pregnancy, sociodemographic characteristics (age, educational attainment, race/ethnicity, and household income), social norms, and knowledge/attitude regarding breastfeeding influence a woman’s intent to breastfeed [22–24]. Immediately following childbirth, women make choices regarding whether to breastfeed their infant or not. Prenatal intent to breastfeed and delivery at a baby-friendly hospital are important contributors to breastfeeding initiation at this stage. After returning home from the hospital, women need support from health professionals, family members (especially the partner) and the workplace to overcome barriers (such as lactation problems and returning to work) to maintenance of breastfeeding throughout the first 6 months [25–27].

### Model specification

The ABM models breastfeeding intention and experiences of the primiparous cohort at three stages: prenatal, childbirth and postpartum. The model simulates breastfeeding decisions and experiences of each woman over a period of 6 months, i.e., from the end of her pregnancy to 6 months after childbirth, during which she may experience common barriers to breastfeeding and may access various breastfeeding promotion interventions.

### Agents

There is only one type of agent in this model: women (expectant mothers and mothers). Women are ‘endowed’ with sociodemographic characteristics that influence their breastfeeding practices, including age (in years), educational attainment (less than high school, or high school graduate or above), race/ethnicity (Hispanic or Non-Hispanic), and household income (≤100% of federal poverty level or >100% of federal poverty level).

### Agent behaviors

**Fig 2:**
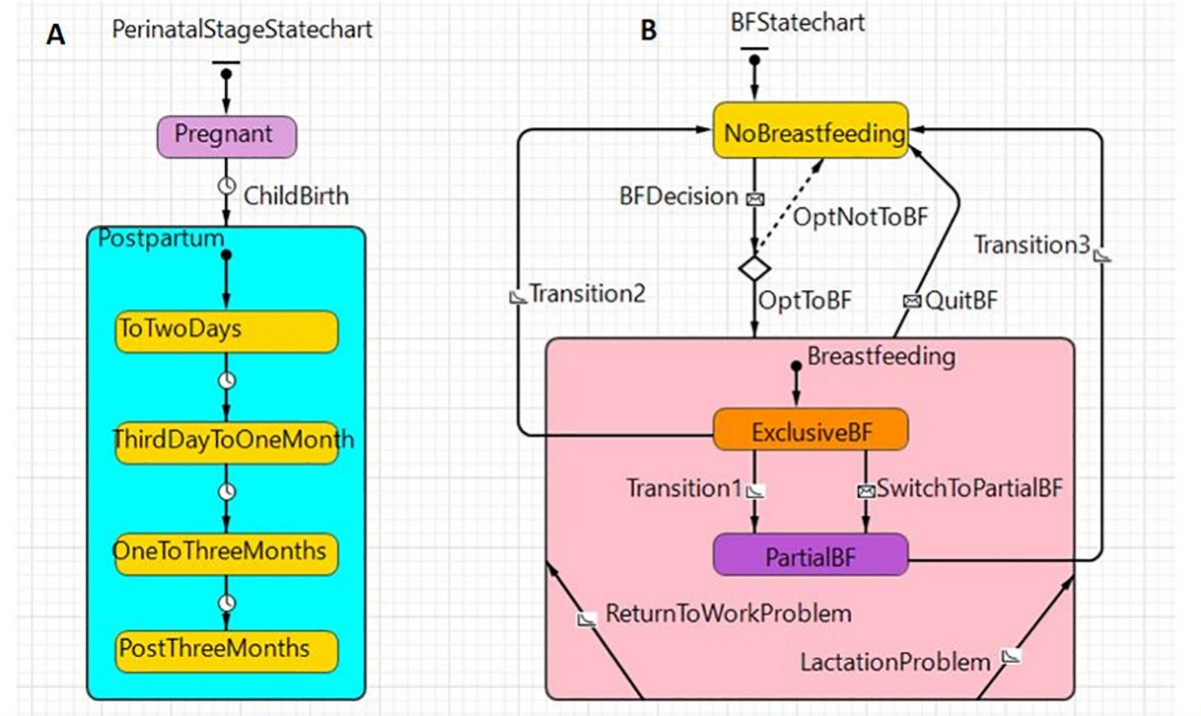
The perinatal stage state chart (A) and breastfeeding status state chart (B).

Women’s breastfeeding decisions and status (intent to breastfeed, initiation of breastfeeding, exclusive breastfeeding, partial breastfeeding and no breastfeeding) were captured using state charts (Fig 2). A state chart shows the state space (the possible states), the events that cause a transition from one state to another, and the actions that result from state change. The **perinatal stage state chart** (Fig 2A) reflects the various stages, determined by time, through which each pregnant woman progresses, including pregnancy, childbirth and postpartum stage. At each step in the perinatal process, women make decisions about their infant feeding options. During pregnancy, they form their intent to breastfeed or formula-feed based on their sociodemographic characteristics and breastfeeding knowledge. During their hospital stay, usually ranging from 1–2 days after childbirth, they decide whether to initiate breastfeeding their infant or not. After returning home from hospital, from the third day to six months postpartum, they encounter support (such as counselling service by health professionals and encouragement from family members) as well as barriers to breastfeeding (including lactation problems and having to return to work), and make decisions as to whether to continue breastfeeding or not. The **breastfeeding status state chart** (Fig 2B) represents the dynamics of these infant feeding options for each woman during the postpartum stage. Women change their breastfeeding status probabilistically with a specified transition rate or when they encounter barriers to breastfeeding such as lactation problems or having to return to work. The decision-making process for each woman when she encounters lactation problems or has to return to work was modeled using decision trees (S1 Fig and S2 Fig in the S1 Supplement). For example, when a lactation problem arises, a decision tree is used to determine how a woman would decide whether to continue to breastfeed or not. In our ABM, we assume that this decision depends on whether she has support from a professional lactation consultant and/or partner. If she has support from both a lactation consultant and her partner, she will continue to breastfeed as she has been doing (exclusively or partially). If she has support from only one source, she will switch from exclusive breastfeeding to partial breastfeeding or from partial breastfeeding to formula feeding. Three transition rates are included in the breastfeeding state chart to account for other reasons for transition from exclusive to partial to no breastfeeding.

The following five breastfeeding promotion interventions were randomly assigned to agents based on the estimates of exposure derived from literatures and empirical data: (1) breastfeeding education, such as prenatal breastfeeding counseling, to increase breastfeeding knowledge, the effectiveness indicated by a score of breastfeeding knowledge ranging from 0 (little knowledge) to 1 (perfect knowledge); (2) Baby-Friendly Hospital Initiative practices, indicated by birth at a designated Baby-Friendly facility (Yes/No); (3) postpartum breastfeeding counselling, indicated by having access to a lactation consultant after childbirth (Yes/No); (4) strengthening partner support, indicated by living with a partner (Yes/No); and (5) fostering supportive workplace environment, indicated by the workplace providing accommodations for nursing women to maintain breastfeeding such as break time and private rooms for pumping breastmilk (Yes/No).

### Network

To calculate the probability of a woman making a particular decision regarding breastfeeding, it was assumed that this decision was based on innate characteristics (e.g. age, education, race/ethnicity) as well as a network of interactions among agents (women) when they form their prenatal breastfeeding intentions and when they need to make infant feeding decisions in the postpartum stage. In formulating this network, we assume that women prefer to network with other women of the same race/ethnicity and that their decisions are influenced by this network of interaction.

### Outcome measures

Primary model outcome measures are the prevalence of breastfeeding intention, the incidence of breastfeeding initiation, and breastfeeding rates (‘any’ and ‘exclusive’) at 1 month, 3 months and 6 months postpartum. ‘Any’ breastfeeding was defined as the child having ever been fed breast milk; ‘exclusive’ breastfeeding was defined as the child having been fed no foods or liquids other than breast milk, not even water [1, 28].

### Parameterization

The 2014 Los Angeles County WIC Survey (lawicdata.org/survey) provided the socio-demographic data needed to simulate the cohort for the ABM. All data obtained from the LA County WIC survey were provided in an anonymized format. Fifty-three records in the survey data set were excluded when data on any one of the four sociodemographic characteristics were missing; a total of 4,646 records were included for this study. We randomly selected 75% of the included records (n = 3,845) into a training sample to simulate the agent population and calibrate the model; the remaining 25% (n = 1,161) were used as a testing sample to validate the model. Since socio-demographic characteristics are often correlated with each other, individual-level data (rather than aggregated distributional data) were used in the model to preserve the correlation among sociodemographic variables and reflect the actual heterogeneity of agents. Data from this 2014 WIC survey were used, together with information from a review of the literature on the effects of selected intervention strategies on breastfeeding practices [29], to estimate parameter values needed for building the ABM. Information to estimate the occurrence of other relevant behaviors such as when women return to work and lactation problems were similarly obtained [30]. A summary of values and data sources of the key parameters used in the model is given in Table 1. Additional details are provided in the supplement.

**Table 1:** Data sources and values for key parameters.

Parameters	Distribution of initial values	Data source or references
**Socio-demographics**		
Age	28.1±6.4 (mean ± SD)	WIC 2014 survey
Education	• Less than high school: 36.5%	WIC 2014 survey
	• High school graduate or above: 64.5%	
Household income	• ≤100% federal poverty level: 48.4%	WIC 2014 survey
	• >100% federal poverty level: 51.6%	
Race/ethnicity	• Hispanic: 85.0%	WIC 2014 survey
	• Non-Hispanic: 15.0%	
**Occurrence of barriers to breastfeeding maintenance**		
Lactation problems	• 0–6 months: 87.4%	Februhartanty, Bardosono and Septiari [30]
Return to work	• 0–2 months: 9.1%	WIC 2014 survey
	• 3–5 months: 14.4%	
	• ≥ 6 months: 13.0%	
	• Not employed: 63.5%	
**Baseline coverage of interventions**		
BF knowledge	Beta-distribution (Mean: 0.67, SD: 0.10, Range: 0–1)	Mitra et al. [29]
Baby-Friendly Hospital Initiative practices	11.4%	WIC 2014 survey
Postpartum breastfeeding counselling	78.1%	WIC 2014 survey
Postpartum partner support	67.7%	WIC 2014 survey
Supportive workplace environment	52.1%	WIC 2014 survey
**Intervention effect**		
Improving BF knowledge on breastfeeding intention	Logistic regression coefficient: 1.17	Mitra et al. [29]
Baby-Friendly Hospital Initiative practices on breastfeeding initiation	Logistic regression coefficient: 0.155	WIC 2014 survey

### Calibration and validation of the model

We included three transition rate parameters in the model to account for residual reasons for discontinuing breastfeeding other than lactation problems and returning to work. The parameter values for these three transition rates–(1) transition from exclusive breastfeeding to partial breastfeeding, (2) transition from partial breastfeeding to formula feeding, and (3) transition from exclusive breastfeeding to formula feeding–were determined by calibration. Specifically, the simulated outcomes (exclusive and any breastfeeding rates at 1, 3 and 6 months postpartum) were compared with the observed rates from the training sample data, using the root mean square error (RMSE); parameter values of the model which best replicated the observed outcomes were selected [31].

### Experiments

We ran experiments that involved increasing the coverage level of each of the five breastfeeding interventions (breastfeeding education, Baby-Friendly Hospital Initiative practices, postpartum breastfeeding counselling, strengthening partner support, and fostering supportive workplace environment) from baseline to three different levels (i.e. 80%, 90% and 95%) while keeping the coverage of other interventions at the baseline level. We also ran scenarios in which several interventions were implemented simultaneously as a “package”. Each experiment was run 100 times with random seeds. For each run, we recorded the proportion of women who initiated breastfeeding and proportions with any or exclusive breastfeeding at 1, 3 and 6 months postpartum. The predicted breastfeeding rates were compared across scenarios to identify the most effective interventions for breastfeeding promotion in this population.

### Sensitivity analysis

Sensitivity analyses were conducted to test how varying parameter values of the intervention effect of the selected intervention strategies might affect the simulation results. Two parameters were selected for sensitivity analysis: the intervention effect of breastfeeding education on prenatal breastfeeding intention, and the intervention effect of the Baby-Friendly Hospital Initiative practices on breastfeeding initiation. Estimates for these parameters were not available from randomized control trials, and were derived from the literature and the WIC 2014 survey data.

## Results

### Model calibration and validation

Our calibration produced a combination of best fit parameter values of the three transition rates (0.016, 0.059 and 0.139 per month), as shown in Table 2.

**Table 2:** Best fit parameter values from model calibration.

Parameters for calibration	Initial value	Value tested in calibration	Final value
Transition rate from exclusive breastfeeding to formula feeding	0.018	0.004–0.08	0.016
Transition rate from partial breastfeeding to formula feeding	0.113	0.02–0.5	0.059
Transition rate from exclusive breastfeeding to partial breastfeeding	0.069	0.015–0.3	0.139

Transition rate in this table refers to the proportion of woman who transition from one infant feeding state to another per month.

**Fig 3:**
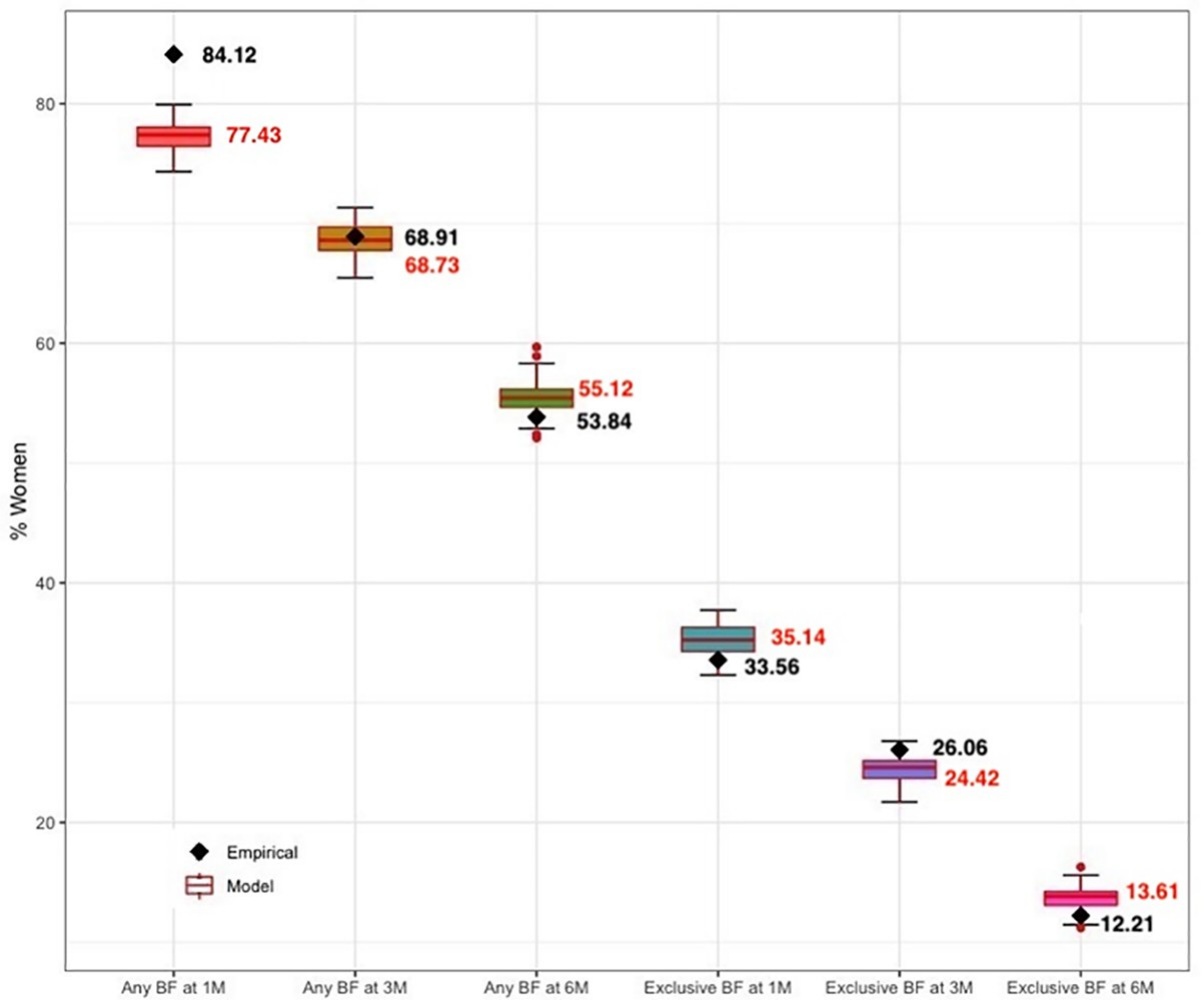
Comparison of model generated breastfeeding rates (boxplot) and observed breastfeeding rates from the testing sample (black diamond) at 1 month, 3 months and 6 months postpartum for the baseline scenario.

Using the calibrated values, we investigated whether the model could reproduce the observed outcomes in the testing sample. Fig 3 shows that except for the any breastfeeding rate at 1 month postpartum, all other breastfeeding rates generated by the model fitted well with the empirical data (RMSE = 3.84). Therefore, we used this validated model with the combination of parameter values to run the experiments.

### Experiments

Increasing the coverage of the selected interventions improved the breastfeeding rates in the cohort to various extents. Table 3 summarizes the predicted breastfeeding rates in scenarios with various hypothetical coverage levels of each of the five interventions described above. For each experiment, the results were calculated based on the average values of 100 runs to reduce stochastic variability.

**Table 3:** Breastfeeding rates predicted at various coverage levels of five selected interventions.

Interventions and their coverage levels	Mean prevalence of breastfeeding practices (%)							
	BF intention	BF initiation	Any BF at 1 month	Any BF at 3 month	Any BF at 6 month	Exclusive BF at 1 month	Exclusive BF at 3 month	Exclusive BF at 6 month
**Improving breastfeeding knowledge**	** **	** **	** **					
Base level (0.67)	88.50	92.96	77.41	68.86	55.62	35.20	24.46	13.86
Intervention level 1 (0.80)	90.05	93.42	77.82	69.20	55.84	35.47	24.64	13.90
Intervention level 2 (0.90)	91.01	93.72	77.99	69.48	56.11	35.59	24.68	13.94
Intervention level 3 (0.95)	91.37	93.84	78.03	69.46	56.24	35.53	24.74	14.03
**Baby-Friendly Hospital Initiative practices**								
Base level (11.40%)	88.50	92.96	77.41	68.86	55.62	35.20	24.46	13.86
Intervention level 1 (80%)	88.52	93.61	77.96	69.44	56.25	35.50	24.71	13.96
Intervention level 2 (90%)	88.67	93.67	77.99	69.49	56.10	35.65	24.69	13.99
Intervention level 3 (95%)	88.66	93.71	77.89	69.37	56.04	35.54	24.65	13.93
**Postpartum professional counseling**								
Base level (78.12%)	88.50	92.96	77.41	68.86	55.62	35.20	24.46	13.86
Intervention level 1 (80%)	88.65	92.94	77.58	69.20	56.03	35.52	24.69	13.91
Intervention level 2 (90%)	88.52	92.98	78.35	70.03	56.80	36.48	25.48	14.39
Intervention level 3 (95%)	88.64	93.02	78.62	70.32	57.09	36.86	25.59	14.65
**Postpartum partner support**								
Base level (67.74%)	88.50	92.96	77.41	68.86	55.62	35.20	24.46	13.86
Intervention level 1 (80%)	88.73	93.04	78.92	70.59	57.33	37.04	25.78	14.62
Intervention level 2 (90%)	88.58	93.05	80.16	71.88	58.71	38.36	26.84	15.31
Intervention level 3 (95%)	88.54	92.96	80.69	72.47	59.53	39.23	27.48	15.75
**Supportive workplace environment**								
Base level (52.05%)	88.50	92.96	77.41	68.86	55.62	35.20	24.46	13.86
Intervention level 1 (80%)	88.64	93.08	77.54	69.82	58.01	35.32	25.01	14.68
Intervention level 2 (90%)	88.58	93.06	77.83	70.30	58.98	35.74	25.35	15.11
Intervention level 3 (95%)	88.64	93.08	77.75	70.31	59.28	35.61	25.25	15.14

Improving the breastfeeding-related knowledge/attitude score from the baseline level (mean: 0.67) to the highest level (mean: 0.95) through education increased the percentage of pregnant women who intend to breastfeed from 88.5% to 91.3%, but conferred only minimal positive impact on the breastfeeding initiation rate and any breastfeeding and exclusive breastfeeding rates in the postpartum period. Increasing the coverage of the Baby-Friendly Hospital Initiative practices led to an increase in the breastfeeding initiation rate (from 93.0% to 93.7%), and only a very slight increase in any breastfeeding and exclusive breastfeeding rates in the later postpartum period. Increasing coverage of the other three postpartum interventions had no impact on breastfeeding intention or initiation but helped to maintain breastfeeding and significantly improved long-term breastfeeding rates. For example, increasing the coverage of partner support from 67.7% (baseline) to 95% leads to an increase in any breastfeeding rate at 6 months, from 55.6% to 59.5%; and exclusive breastfeeding rate at 6 months, from 13.9% to 15.8%.

Table 4 presents the effects of various intervention packages (made up of multiple interventions) on breastfeeding rates. Compared to scenarios with a single intervention, increasing the coverage levels of multiple interventions included in an intervention package significantly improved breastfeeding rates, particularly in the postpartum period. For example, when the coverage of four interventions delivered in a package (improving breastfeeding knowledge, Baby-Friendly Hospital Initiative practices, postpartum breastfeeding counseling, and strengthening partner support) was increased from the baseline level to intervention level 3 (95%), the predicted any breastfeeding rate at 6 months increased by 8.8 percentage points from 55.6% to 64.4%. In comparison, the sum of the effects of these four interventions when delivered separately amounts to only 6.4 percentage points.

**Table 4:** Breastfeeding rates predicted at various coverage levels of selected intervention packages.

Intervention packages and their coverage levels	Mean prevalence of breastfeeding practices (%)							
	BF intention	BF initiation	Any BF at 1 month	Any BF at 3 month	Any BF at 6 month	Exclusive BF at 1 month	Exclusive BF at 3 month	Exclusive BF at 6 month
**KNWL**	** **	** **	** **					
Base level	88.50	92.96	77.41	68.86	55.62	35.20	24.46	13.86
Intervention level 1 (0.80)	90.05	93.42	77.82	69.20	55.84	35.47	24.64	13.90
Intervention level 2 (0.90)	91.01	93.72	77.99	69.48	56.11	35.59	24.68	13.94
Intervention level 3 (0.95)	91.37	93.84	78.03	69.46	56.24	35.53	24.74	14.03
**KNWL+BHFI**								
Base level	88.50	92.96	77.41	68.86	55.62	35.20	24.46	13.86
Intervention level 1 (0.80)	90.08	93.95	78.26	69.66	56.27	35.64	24.69	13.93
Intervention level 2 (0.90)	91.07	94.36	78.41	69.82	56.43	35.73	24.78	14.04
Intervention level 3 (0.95)	91.36	94.44	78.68	70.09	56.60	35.99	25.07	14.17
**KNWL+BHFI+COUL**								
Base level	88.50	92.96	77.41	68.86	55.62	35.20	24.46	13.86
Intervention level 1 (80%)	90.00	94.02	78.33	69.84	56.42	35.95	25.02	14.12
Intervention level 2 (90%)	90.91	94.27	79.41	70.89	57.49	36.87	25.74	14.55
Intervention level 3 (95%)	91.47	94.45	79.90	71.55	58.22	37.44	26.15	14.86
**KNWL+BHFI+COUL+PTR**								
Base level	88.50	92.96	77.41	68.86	55.62	35.20	24.46	13.86
Intervention level 1 (80%)	89.98	93.88	79.94	71.60	58.29	37.75	26.38	14.94
Intervention level 2 (90%)	91.00	94.36	83.41	75.39	62.24	41.67	29.47	17.05
Intervention level 3 (95%)	91.37	94.46	85.16	77.25	64.38	44.43	31.37	18.22
**KNWL+BHFI+COUL+PTR+WP**								
Base level	88.50	92.96	77.41	68.86	55.62	35.20	24.46	13.86
Intervention level 1 (80%)	89.92	93.93	80.07	72.39	60.60	38.01	27.04	16.04
Intervention level 2 (90%)	90.89	94.33	83.64	76.50	65.47	42.24	30.49	18.51
Intervention level 3 (95%)	91.34	94.49	85.49	78.66	68.19	44.69	32.45	20.05

KNWL: improving breastfeeding knowledge; BFHI: Baby-Friendly Hospital Initiative practices; COUL: postpartum breastfeeding counselling; PTR: strengthening partner support; and WP: fostering supportive workplace environment.

### Sensitivity analysis

Table 5 summarizes the results of sensitivity analyses with varying values of the two parameters, the intervention effect of improving breastfeeding knowledge on prenatal breastfeeding intention (logistic regress coefficients ranging from 1.0 to 1.5), and the intervention effect of the Baby-Friendly Hospital Initiative practices on breastfeeding initiation (logistic regression coefficients ranging from 0.1 to 0.5). The predicted breastfeeding rates with varying values of the two parameters were not significantly different when the coverage of each intervention was set at 95%. Therefore, varying the values of these two parameters did not have a major impact on the breastfeeding rates predicted by the ABM (Table 5).

**Table 5:** Predicted breastfeeding rates with 95% intervention coverage: Sensitivity analysis.

Parameters and their values	Breastfeeding outcomes with universal intervention coverage (95%)							
	BF intention	BF initiation	Any BF at 1 month	Any BF at 3 month	Any BF at 6 month	Exclusive BF at 1 month	Exclusive BF at 3 month	Exclusive BF at 6 month
**Causal effect of breastfeeding knowledge/attitude on breastfeeding intention **								
1.0	91.07	93.75	78.05	69.54	56.15	35.55	24.75	13.92
1.17 (value in the model)	88.50	92.96	77.41	68.86	55.62	35.20	24.46	13.86
1.25	91.66	93.92	78.14	69.61	56.21	35.38	24.50	13.86
1.5	91.89	94.03	78.09	69.48	56.22	35.62	24.76	13.94
**Causal effect of Baby-Friendly Hospital Initiative practices on breastfeeding initiation **								
0.1	88.67	93.06	77.30	68.89	55.64	35.38	24.62	13.90
0.155 (value in the model)	88.50	92.96	77.41	68.86	55.62	35.20	24.46	13.86
0.25	88.51	93.07	77.46	68.95	55.70	35.36	24.55	13.86
0.5	88.65	93.23	77.49	68.96	55.59	35.30	24.45	13.74

## Discussion

The agent-based model that we developed replicated the empirical data and helped predict individual- and population-level intervention effects. The effects on breastfeeding practices were different for the five selected interventions among WIC participants.

Among the five individual interventions, increasing coverage of postpartum professional counseling, partner support and supportive workplace environment led to significant improvement in breastfeeding maintenance, i.e., breastfeeding rates at 1 month, 3 months and 6 months postpartum. Since it is common for nursing women to encounter lactation problems, support from health professionals and family members is critical for helping mothers succeed in breastfeeding maintenance. Surprisingly, having a supportive workplace environment showed a positive impact on breastfeeding rates in this population of WIC-enrolled even though only a quarter of women returned to work within 6 months postpartum. This finding supports the efforts of the WIC program to provide breastfeeding support to moms who return to work (e.g. provision of pumps, outreach to employers). For populations where the majority of women return to work soon after childbirth, we might expect an even larger impact of fostering supportive workplace environment. In the United States, where employers are required to provide only unpaid maternity leave of up to 12 weeks to certain eligible workers [32], workplace barriers to breastfeeding (such as the lack of space for pumping breastmilk) must be addressed to support breastfeeding maintenance.

Improving prenatal breastfeeding knowledge/attitude had a positive impact on women’s intent to breastfeed but not on postpartum breastfeeding outcomes. This result is consistent with a meta-analysis by Guise et al. [33] which found that education programs to improve knowledge/attitude are effective in increasing breastfeeding initiation rates, but have no significant effects on long-term breastfeeding duration. Similarly, increasing coverage of delivery at baby-friendly hospitals improves breastfeeding initiation rate but achieves only modest increases in long-term breastfeeding rates. This finding differs from that of a cluster randomized trial conducted in the Republic of Belarus by Kramer et al. [34]. In that study, Baby-Friendly Hospital Initiative practices were found to be effective in increasing both duration and exclusivity of breastfeeding. However, two unique features of the Belarussian health care system–high centralization and prolonged postpartum hospital stay for childbirth–may explain their finding of a larger intervention effect.

The comparative effectiveness of these interventions also reflects a common feature of complex systems–that of path dependence [35, 36]. The path dependence feature means that the dynamic process is contingent, non-reversible and evolutionary based on its own history [36]. The fact that breastfeeding cannot resume once it is interrupted for more than a few days puts a premium on achieving breastfeeding maintenance uninterrupted throughout the entire post-partum period, across many months. Therefore, postpartum professional counseling and partner support and supportive workplace environment play a critical role in maintaining breastfeeding behavior *all through the postpartum period*. In comparison, the other two interventions, education and Baby-Friendly Hospital Initiative practices, occur at specific periods, in the prenatal period and the immediate period following delivery respectively.

Increasing coverage of multiple interventions simultaneously improved the predicted breastfeeding rates significantly for the postpartum period. The results reflect the synergistic effect of combinations of interventions in a complex system where the whole is greater than the sum of the parts. In this case, the combined effect of multiple breastfeeding promotion interventions is greater than the sum of the individual effect of each intervention.

Application of the agent-based modeling methods in this study brings some advantages over previous research that employed traditional variable-based regression methods. First, this ABM model is dynamic in character. In contrast to the more static regression-based approaches, this key feature allows us to portray the dynamic process of articulated decision-making or evolution of social networks relevant to a woman’s infant feeding behaviors within the first six month postpartum. Second, the modeling of each individual woman’s decision-making process allows us to incorporate behavioral theories such as the Theory of Planned Behavior [37, 38] and Social Cognitive Theory [39] in the model, which facilitates our understanding of how these theories work together to predict/explain a behavioral outcome. Third, it is relatively easy to capture the interactions between individuals (through network effects) and between individuals and their environment (e.g., individuals’ contact with health care system and workplace) in the simulation model. Fourth, an ABM allows us to explore the behavioral dynamics at the individual level and to assess the effect of interventions at the aggregated population level so that the population-level effect of certain interventions can be obtained directly from the simulation results. Finally, agent-based modeling enables us to run experiments with intervention coverage set at any level, thereby offering richer information for policy makers to weigh alternative intervention options.

There are limitations to this study. First, it was difficult, even impossible in some cases, to extract appropriate parameter values from the literature, since randomized control trials were not available for all parameter estimates. Therefore, we derived some parameter values (e.g., the effect of improving breastfeeding knowledge on breastfeeding intention) from observational studies, which may be biased. For the effect of the three postpartum interventions (breastfeeding counseling, strengthening partner support and fostering workplace supportive environment) for which there is lack of literature to inform the selection of parameter estimates [40], there is the possibility that the intervention effect size may be overestimated. Future research is needed to fill this gap by conducting more rigorous experimental/quasi-experimental studies or applying causal inference methods to generate more robust estimates from existing data. Second, we selected five breastfeeding promotion interventions that are feasible for our target population but this list of interventions is neither exhaustive nor complete. For example, a number of WIC clinics provide *peer counseling* for nursing women but we were not able to include this specific form of counselling as an intervention due to the lack of information on the extent of its use. Data on the coverage of these and other interventions will allow future studies to assess the effects of these interventions. Finally, the conceptualization of this ABM relied mainly on the research team and expert input. Although we made efforts to incorporate opinions from lactation consultants who serve the WIC population, we were not able to involve the target population directly in the conceptualization process due to resource and logistical constraints. It will be useful for future studies to incorporate nursing women’s perspectives and experiences in the development of the ABM to improve understanding of the behavioral decision process as it affects the effectiveness of interventions.

## Conclusion

Agent-based modeling is a useful tool for understanding the dynamic process of decision-making regarding the effectiveness of various behavioral interventions in a vulnerable low-income population; the use of a socio-ecological framework further allowed the consideration and inclusion of environmental policy interventions. To our knowledge, this is the first study to use agent-based modeling to examine breastfeeding practices and the potential impact of various interventions. By allowing the consideration of many levels of risk and protective factors, and their dynamic interactions, agent-based modeling provides a tool for bringing together decision-makers to understand the population impact of various intervention strategies.

## Supporting information

S1 Supplement (DOCX)
