# Supplementary material for: Estimating the population impact of hypothetical breastfeeding interventions in a low-income population in Los Angeles County: An agent-based model
Source: PLoS One. 2020 Apr 9;15(4):e0231134. doi: 10.1371/journal.pone.0231134 (PMC7145098; doi:10.1371/journal.pone.0231134)
Supplement: S1 Supplement — (DOCX) [file pone.0231134.s001.docx]

**Supplement**

This supplementary material details the agent-based modeling methods for the paper, “Estimating the population impact of hypothetical breastfeeding interventions in a low-income population in Los Angeles County: An agent-based model” by Linghui Jiang, Xiaoyan Li, May C. Wang, Nathaniel Osgood, Shannon E. Whaley, and Catherine M. Crespi.

In this paper, we developed an agent-based model (ABM) to simulate the breastfeeding experience of a cohort of primiparous women during the first six months postpartum. Then we used the model to predict the prevalence of breastfeeding practices under various scenarios with different coverage levels of breastfeeding promotion interventions.

1. Model structure

Using the simulation software AnyLogic (version 8.3.2), we developed a non-spatial agent-based model in which we simulated a cohort of primiparous women with different sociodemographic characteristics and modeled their breastfeeding experience during the first six months postpartum. Each woman enters the model on the last day of their pregnancy and spends six months in the model. The model time unit is one month. The model operates on discrete time steps and the time step for solving equations is 0.001 month.

The ABM models breastfeeding intention and experiences of this primiparous cohort at three stages, prenatal, childbirth and postpartum, using two state charts and two decision trees. State charts are state transition diagrams that identify states and describe event- and time-driven transitions from one state to another. One state chart (Fig 2A) is used to specify perinatal stage (prenatal, childbirth, postpartum) and the duration of each stage, and the other (Fig 2B) is used to represent women’s breastfeeding status in the childbirth and postpartum stages (exclusive, partial and no breastfeeding) and transitions between these breastfeeding states.

Breastfeeding intention is formed at the prenatal stage and calculated on the last day of pregnancy based on a woman’s sociodemographic characteristics, breastfeeding knowledge score and the influence of her peers. Once her child is born, the woman makes a decision about whether or not to initiate breastfeeding based on her prenatal breastfeeding intention and access to Baby-Friendly Hospital Initiative services. If breastfeeding is initiated, it begins as exclusive breastfeeding. Two major barriers to breastfeeding may occur, lactation problems and having to return to work, in the first six months postpartum. These barriers occur probabilistically. When encountering one of these barriers, the woman may assess the situation and adjust her breastfeeding status which was modeled using a decision tree. A decision tree is a tree-like graph that depicts a sequence of decisions and their possible consequences. Three transition rates are included in the breastfeeding status state chart to account for other reasons that women transition from exclusive to partial to no breastfeeding. In the model, each woman is also connected with some of her peers and her breastfeeding intention and decisions are influenced by her interactions with peers.

1. Agent attributes and assignment of initial values

Pregnant women are the agents in this model. The 2014 Los Angeles County WIC survey data (lawicdata.org/survey) informed the simulation of the agent population (primiparous women). The agents were associated with both fixed attributes (sociodemographic characteristics) and modifiable attributes (experiencing barriers to breastfeeding, and access to breastfeeding promotion interventions). This section provides the definition of each attribute and describes how the initial values of the attributes are assigned.

- 1. Sociodemographic characteristics

Four sociodemographic characteristics of agents (pregnant women) were defined, age, race/ethnicity, educational attainment and household income. Table S-1 provides description of each sociodemographic characteristic attribute. Age is a numerical variable and the other three attributes (race/ethnicity, educational attainment and household income level) are categorical, consistent with the operationalization of these variables in the 2014 Los Angeles County WIC survey (<http://lawicdata.org/survey/>).

Individual-level data from the 2014 Los Angeles County WIC survey were imported to assign the initial values of sociodemographic characteristics to each agent. Fifty-three records were excluded due to missingness on any one of the four sociodemographic characteristics; a total of 4,646 records from the survey data were included. We randomly selected 75% of the included records (n=3,845) into a training sample to simulate the agent population and calibrate the model; the remaining 25% records (n=1,161) were used as a testing sample to validate the model. Although age and household income may change with time, the duration of our model is only 6 months during which the changes are most likely ‘ignorable’ and will not have an impact on the model output. We thus model all four of the sociodemographic characteristics including age and household income as fixed attributes, i.e., the values of these attributes do not change with time. The distributions of the initial values for the sociodemographic characteristics are presented in table S-1.

Table S-1 Sociodemographic characteristics of agents and distribution of the initial values

| **Attribute** | **Operational definition** | **Distribution of initial values** | **Data source** |
| --- | --- | --- | --- |
| Age | Women’s age at childbirth, continuous variable (years) | 28.1±6.4 (mean ± SD) | WIC 2014 survey training sample |
| Education | Women’s highest educational attainment, categorical variable | - Less than high school: 36.5% - High school graduate or above: 64.5% | WIC 2014 survey training sample |
| Household income | Household income relative to federal poverty level (FPL), categorical variable | - ≤100% FPL: 48.4% - >100% FPL: 51.6% | WIC 2014 survey training sample |
| Race/ethnicity | Self-reported race/ethnic groups, categorical variable | - Hispanic: 85.0% - Non-Hispanic: 15.0% | WIC 2014 survey training sample |

- 1. Network

There is evidence that peer influence plays an important role in women’s decision making about infant feeding methods [1-4]. In this ABM, each woman was connected with an average of three peers. The number of connected peers followed a truncated normal distribution N (3, 1^2^) with a minimum value of 0 and a maximum value of 6. The greatest integer function “floor ()” was used to return an integer from the distribution for each agent which specifies the number of their connected peers. The mean number of connected peers was derived from a study by Buckner and Matsubara [5]. For each agent, which peers to connect with depends on a preference score based on race/ethnicity. Peers from the same racial/ethnic group were assigned higher preference scores.

- 1. Experiencing barriers to breastfeeding maintenance

After initiating breastfeeding, some women may encounter barriers to maintaining breastfeeding. Experiencing lactation problems and having to return to work are the two major reasons for early termination of breastfeeding throughout the first 6 months postpartum [6-8]. Agents are randomly assigned to experience these two problems each month. The proportion of women who may experience each problem varies by month. The rates of occurrence of lactation problems and returning to work are based on the study by Februhartanty, Bardosono and Septiari [9] and the 2014 WIC survey data, respectively. The definitions of the two problems and the estimated percentages of women experiencing each problem are provided in Table S-2.

Table S-2 Barriers to breastfeeding maintenance and assignment of the initial values

| **Attribute** | **Operational definition** | **Occurrence in each month** | **Data source** |
| --- | --- | --- | --- |
| Experiencing lactation problems | Whether a mother experienced any lactation problems during the first 6 months postpartum (Yes/No) | - 1^st^ month: 70% - 2^nd^ month: 3.4% - 3^rd^ month: 3.4% - 4^th^ month: 3.4% - 5^th^ month: 3.4% - 6^th^ month: 3.4% | Februhartanty, Bardosono and Septiari [9] |
| Having to return to work | Whether a mother has to return to work during the first 6 months postpartum (Yes/No) | - 1^st^ month: 3% - 2^nd^ month: 3% - 3^rd^ month: 3% - 4^th^ month: 4.8% - 5^th^ month: 4.8% - 6^th^ month: 4.8% | WIC 2014 survey |

- 1. Access to breastfeeding promotion interventions

We examined the effects of women’s access to five categories of breastfeeding promotion interventions in this model (Table S-3): 1) educational interventions, such as prenatal breastfeeding counseling, to increase breastfeeding knowledge, indicated by a score of breastfeeding knowledge ranging from 0 (little knowledge) to 1 (perfect knowledge); 2) Baby-Friendly Hospital Initiative practices, indicated by birth at a designated Baby-Friendly facility; 3) postpartum breastfeeding counselling, indicated by having a consultant or a telephone number to call for help after childbirth; 4) support from partner for breastfeeding, indicated by living with her partner; and 5) supportive workplace environment, indicated by the workplace providing accommodations for nursing women to maintain breastfeeding such as a break time and a lactation room.

The baseline coverage of these interventions was informed by literature review and the 2014 WIC survey data. Each agent was randomly assigned a knowledge score ranging from 0 to 1 which followed a Beta distribution. The study by Mitra et al [10] informed the estimation of the mean (0.67) and standard deviation (0.10) of breastfeeding knowledge; and these values were used to compute the parameters of the Beta distribution. The estimated percentages of agents having access to the other four interventions in baseline scenarios are 11.4%, 78.1%, 67.7% and 52.1, respectively, based on the 2014 WIC survey data. Agents were randomly assigned to access these interventions.

Table S-3 Definition and estimated baseline coverage of breastfeeding promotion interventions

| **Intervention** | **Operational definition of access to the intervention** | **Baseline distribution/ coverage** | **Data source** |
| --- | --- | --- | --- |
| Increasing BF knowledge | A score of breastfeeding related knowledge ranging from 0 (little knowledge) to 1 (perfect knowledge) | Beta distribution  (Mean: 0.67, SD 0.10, range: 0-1) | Mitra et al [10] |
| Baby-Friendly Hospital Initiative practices | Whether a woman gives birth in a designated Baby-Friendly facility (Yes/No) | 11.4% | WIC 2014 survey |
| Postpartum breastfeeding counselling | Whether a woman has a consultant or a telephone number to call for help with breastfeeding (Yes/No) | 78.1% | WIC 2014 survey |
| Postpartum family support | Whether a woman lives with her partner (Yes/No) | 67.7% | WIC 2014 survey |
| Supportive workplace environment | Whether the workplace have accommodations for nursing women to maintain breastfeeding such as a break time or a lactation room (Yes/No) | 52.1% | WIC 2014 survey |

1. Model dynamics and outcomes

This section describes the algorithms and assumptions associated with the model dynamics. The model dynamics pertain to three outcome measures that are assessed in the model: breastfeeding intention at prenatal stage, breastfeeding initiation at childbirth stage, and breastfeeding status at each month during the first six months postpartum.

- 1. Breastfeeding intention

Breastfeeding intention, whether a pregnant woman intents to breastfeed her child or not, was assessed on the last day of pregnancy, when the model starts. It is a dichotomous variable (Yes/No). Based on her sociodemographic characteristics, breastfeeding knowledge score and the influence from her peers, a woman’s initial breastfeeding intention was calculated and assigned in the following four steps.

Step 1: A logistic regression equation was applied to predict probability of intending to breastfeed based on sociodemographic characteristics and breastfeeding knowledge score.

$$P_{int:agent}=\frac{e^{XB}}{1+e^{XB}}= \frac{e^{1.64-0.22Age20-0.07Age2030+0.54Hispanic+0.52HSgrad-0.43Income+1.17(Knowledge-0.67)}}{1+e^{1.64-0.22Age20-0.07Age2030+0.54Hispanic+0.52HSgrad-0.43Income+1.17(Knowledge-0.67)}}$$

Note:

1. Age20: binary variable, 1= age < 20, 0 otherwise; Age2030: binary variable, 1= age 20-30, 0 otherwise; Hispanic: binary variable, 1=Hispanic, 0 otherwise; Hsgrad: binary variable, 1= high school graduate or above, 0 otherwise; income: binary, 1= household income ≤100% Federal Poverty Level, 0 otherwise; Knowledge: continuous variable, values ranging 0-1.
2. The logistic regression coefficients for sociodemographic variables were estimated from the WIC 2014 survey data.
3. The logistic regression coefficient for breastfeeding knowledge was derived from a study by Mitra et al [10].

Step 2: Calculate the mean probability of intending to breastfeed of an agent’s peers with whom she is directly connected.

$$P_{int:peer}=\frac{\sum_{1}^{n} P_{n}}{n}$$

Note:

1. n is the number of an agent’s connected peers
2. P_n_ is each peer’s probability of intending to breastfeed calculated in step 1.

Step 3: Calculate the final probability of intention to breastfeed, which is the average of an agent’s original probability (step 1) and the mean probability of her connected peers (step 2).

$$P_{int}=\frac{P_{int:agent}+P_{int:peer}}{2}$$

Step 4: Using the Bernoulli distribution (in AnyLogic, the randomTrue function), each agent is randomly assigned to intend to breastfeed with probability P_int_ as calculated in step 3.

- 1. Breastfeeding initiation

For each agent, the probability of initiating breastfeeding was calculated immediately after childbirth and an initiation status (Yes/No) was assigned as follows:

Step 1: A logistic regression equation was applied to predict the probability based on breastfeeding intention and delivery at a Baby-Friendly hospital:

$$P_{ini}=\frac{e^{XB}}{1+e^{XB}}= = \frac{e^{0.73+2.50BFint+0.155BFHI}}{1+e^{0.73+2.50BFint+0.155BFHI}}$$

Note:

1. BFint: binary variable, 1= intent to breastfeed, 0 otherwise; BFHI: binary variable, 1=delivery in a Baby-Friendly hospital, 0 otherwise.
2. The logistic regression coefficients were estimated from the WIC 2014 survey data.

Step 2: Using a Bernoulli distribution, each agent is randomly assigned to initiate breastfeeding with probability P_ini_ as calculated in step 1.

- 1. Breastfeeding status during the first six months postpartum

If an agent chooses to initiate breastfeeding after childbirth, she is assumed to start with exclusive breastfeeding. Her breastfeeding status, exclusive breastfeeding, partial breastfeeding or no breastfeeding (formula feeding), is assessed monthly during the first six months postpartum. During this period, some women experience one or two major barriers to breastfeeding, i.e. lactation problems, such as breast engorgement and insufficient milk supply, and having to return to work. When a lactation problem occurs, a nursing woman will go through a decision tree (Figure S-1). Based on her access to professional consultant and family support, the woman may continue to breastfeed, or breastfeed less, or stop breastfeeding. When a nursing woman has to return to work within the first six months postpartum, she will go through another decision tree (Figure S-2). Based on her access to workplace support for breastfeeding, she may continue to breastfeed, or breastfeed less, or stop breastfeeding.


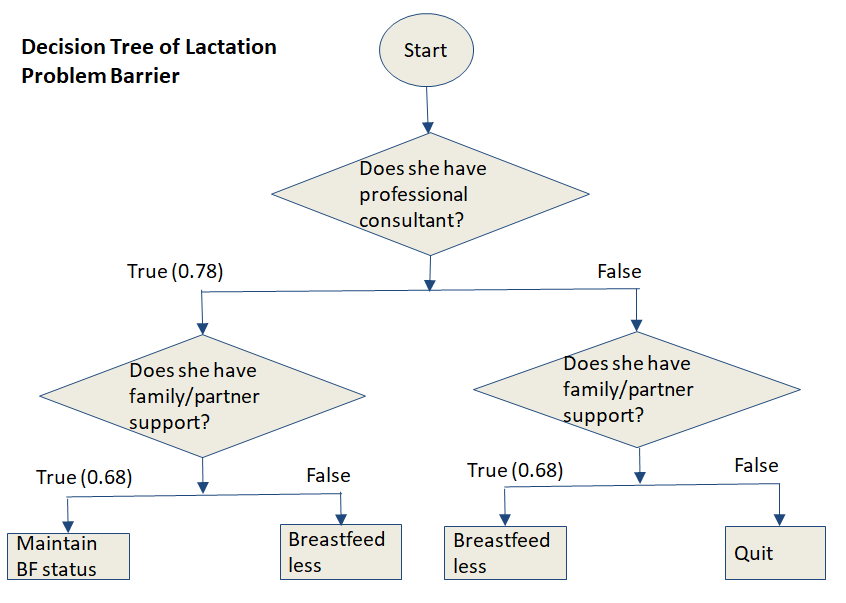

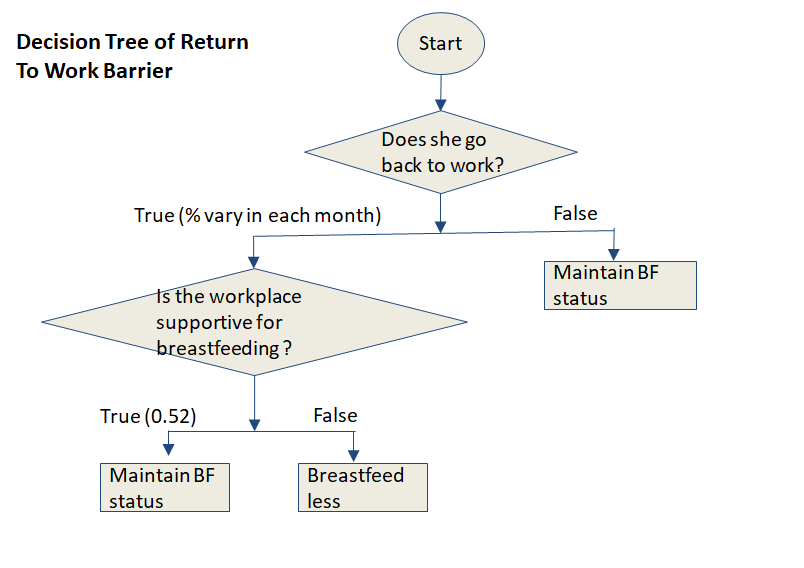


Figure S-1: Decision tree for nursing women experiencing lactation problems

Figure S-2: Decision tree for nursing women who have to return to work

To account for other reasons that women change their breastfeeding status, we included three transition rates in the model, i.e. transition from exclusive breastfeeding to partial breastfeeding (P_EBF→PBF_), transition from exclusive breastfeeding to formula feeding (P_EBF→NBF_), and transition from partial breastfeeding to formula feeding (P_PBF→NBF_). The transition rates in the first month are higher than those in the following months. The estimated transition rates are derived from a study by Jiang et al [11] and we calibrated the values using empirical data.

Table S-4 Three breastfeeding status transition rates and assignment of the initial values

| **Transitions** | **Monthly transition rate** |
| --- | --- |
| Transition from exclusive breastfeeding to partial breastfeeding | - 2^nd^ – 6^th^ month: P_EBF→PBF_ =0.069 - 1^st^ month: 4.6*P_EBF→PBF_ =4.6*0.069=0.32 |
| Transition from exclusive breastfeeding to formula feeding | - 2^nd^ – 6^th^ month: P_EBF→PBF_=0.018 - 1^st^ month: 2.4*P_EBF→PBF_ =2.4*0.018=0.04 |
| Transition from partial breastfeeding to formula feeding | - 2^nd^ – 6^th^ month: P_PBF→NBF_=0.113 - 1^st^ month: 3.4*P_PBF→NBF_ =3.4*0.113=0.38 |

1. Outcomes at the population level

The aggregated outcomes at the population level, measured as the prevalence of breastfeeding intention, the incidence of breastfeeding initiation, and the prevalence of any breastfeeding and exclusive breastfeeding at 1 month, 3 month and 6 months postpartum, were compared with the observed outcomes from the 2014 WIC survey data to validate the model.

**References**

1. Bronner Y, Barber T, Vogelhut J, Resnik AK. Breastfeeding peer counseling: results from the National WIC Survey. J Hum Lact. 2001;17(2):119-25; quiz 32-4, 68. PubMed PMID: 11847825.

2. Campbell LA, Wan J, Speck PM, Hartig MT. Women, Infant and Children (WIC) peer counselor contact with first time breastfeeding mothers. Public health nursing. 2014;31(1):3-9. doi: 10.1111/phn.12055. PubMed PMID: 24387771.

3. Chapman DJ, Damio G, Young S, Pérez-Escamilla R. Effectiveness of breastfeeding peer counseling in a low-income, predominantly Latina population: a randomized controlled trial. Arch Pediat Adol Med. 2004;158(9):897-902.

4. Carlin RF, Mathews A, Oden R, Moon RY. The Influence of Social Networks and Norms on Breastfeeding in African American and Caucasian Mothers: A Qualitative Study. Breastfeeding Medicine. 2019.

5. Buckner E, Matsubara M. Support network utilization by breastfeeding mothers. Journal of Human Lactation. 1993;9(4):231-5.

6. Cross-Barnet C, Augustyn M, Gross S, Resnik A, Paige D. Long-term breastfeeding support: failing mothers in need. Matern Child Health J. 2012;16(9):1926-32. doi: 10.1007/s10995-011-0939-x. PubMed PMID: 22246714.

7. Hedberg IC. Barriers to breastfeeding in the WIC population. MCN Am J Matern Child Nurs. 2013;38(4):244-9. doi: 10.1097/NMC.0b013e3182836ca2. PubMed PMID: 23812063.

8. HHS. The Surgeon General's call to action to support breastfeeding. In: Services USDoHaH, editor. Washington, DC: U.S. Department of Health and Human Services, Office of the Surgeon General; 2011.

9. Februhartanty J, Bardosono S, Septiari AM. Problems during lactation are associated with exclusive breastfeeding in DKI Jakarta Province: father’s potential roles in helping to manage these problems. Malaysian Journal of Nutrition. 2006;12(2):167-80.

10. Mitra AK, Khoury AJ, Hinton AW, Carothers C. Predictors of breastfeeding intention among low-income women. Matern Child Health J. 2004;8(2):65-70. PubMed PMID: 15198173.

11. Jiang L, Nobari TZ, Wang MC, Whaley M, Whaley SE. Examining breastfeeding patterns in the first year among WIC beneficiaries in Los Angeles County [Abstract]. APHA's 2018 Annual Meeting & Expo; Nov. 10 -14; San Diego, CA. 2018.
